# Self-Assembled Serpentine Ni_3_Si_2_O_5_(OH)_4_ Hybrid Sheets with Ammonium Polyphosphate for Fire Safety Enhancement of Polylactide Composites

**DOI:** 10.3390/polym14235255

**Published:** 2022-12-01

**Authors:** Xiaohong Yi, Jingshu Huang, Yizhang Tong, Hui Zhao, Xianwu Cao, Wei Wu

**Affiliations:** 1 Jihua Laboratory, Foshan 528200, China; 2 Key Laboratory of Polymer Processing Engineering of Ministry of Education, South China University of Technology, Guangzhou 510640, China; 3 Guangxi Key Laboratory of Calcium Carbonate Resources Comprehensive Utilization, College of Materials and Chemical Engineering, Hezhou University, Hezhou 542899, China

**Keywords:** polylactide, serpentine, ammonium polyphosphate, composite, flame retardant, mechanical property

## Abstract

Biodegradable polylactide (PLA) has been widely utilized in people’s daily lives. In order to improve the fire safety of PLA, ammonium polyphosphate (APP) was self-assembled onto the surface of serpentine Ni_3_Si_2_O_5_(OH)_4_ through the electrostatic method, followed by mixing with PLA by melt compounding. The APP-modified serpentine (serpentine@APP) dispersed uniformly in the PLA matrix. Compared with pure PLA, the PLA composite with 2 wt% serpentine@APP reduced the peak heat release rate (pHRR) and total heat release (THR) by 43.9% and 16.3%, respectively. The combination of APP and serpentine exhibited suitable synergistic flame-retardant effects on the fire safety enhancement of PLA. In addition, the dynamical rheological tests revealed that the presence of APP and serpentine could reduce the viscosity of PLA composites. The plasticizing effects of APP and serpentine benefited the processing of PLA. The mechanical properties of PLA/serpentine@APP maintained suitable performance as pure PLA. This study provided a feasible way to enhance the fire safety of PLA without sacrificing its mechanical properties.

## 1. Introduction

Biodegradable polymers have drawn considerable attention in both academia and industry due to the increasing awareness of environmental pollution problems. Polylactide (PLA) is a kind of bio-based and biodegradable polyester that shows promising applications in various fields, such as packaging, automotives, and electronics [1,2,3,4]. It exhibits unique properties such as suitable transparency, high mechanical strength, suitable processibility, and non-toxicity. However, the intrinsic flammability of PLA limits its further applications and development. Therefore, it is an important and urgent issue to improve the fire safety performance of PLA [5,6,7]. Recently, flame-retardant PLA composites containing different types of fillers, such as montmorillonite [8], carbon black [9], sepiolite [10], and carbon nanotubes [11], have been extensively investigated. The addition of organic modified montmorillonite (OMMT) enhanced the thermal stability and fire resistance of PLA significantly due to its excellent physical barrier effect and char-forming effect [8]. Wen et al. [9] grafted DOPO onto the surface of carbon black (CB-g-DOPO). The results revealed that CB-g-DOPO was effective at improving the flame retardancy and mechanical properties of PLA/CB composites. The peak heat release rate (pHRR) of the PLA composite containing 10 wt% CB-g-DOPO was reduced by 40.7% as compared with that of pure PLA. Jiang et al. decorated natural sepiolite with DOPO (SEP-g-DOPO) through the reaction between amino groups and salicylaldehyde. The presence of SEP-g-DOPO benefited the generation of the dense and continuous char layer, protecting the interior layer more efficiently during combustion [10].

Two-dimensional (2D) layered materials have exhibited superior flame-retardant behaviors in a variety of polymer composites due to their barrier effect and promotion of char residues [12,13]. Many researchers have explored 2D-based nanofillers in combination with flame-retardant additives to improve the fire performance of PLA [14]. A combination of graphitic carbon nitride (g-C_3_N_4_) with melamine pyrophosphate (MPP) or DOPO exhibited a significant reduction in pHRR and total heat release (THR) of PLA [15]. Xu et al. utilized melamine-cyanuric acid (MCA) hybrids as a shell layer to decorate lamellar molybdenum disulfide (MoS_2_) plates and then mixed them with PLA through melt blending [16]. The core-shell structure of MoS_2_ could suppress the pyrolysis rate and promote the graphitization degree of char residues. Jing et al. demonstrated that the core-shell flame-retardant/graphene oxide (GOH) hybrids could reduce fire hazards and improve the toughness of PLA simultaneously [17]. Zhang et al. reconstructed layered double hydroxides (LDH) with phosphotungstic acid [18]. The PLA composite obtained the UL-94 V-0 rating and achieved a maximal LOI value of 48.3% with 18.0 wt% intumescent flame retardant (IFR) and 2.0 wt% modified LDH. The modified LDH exhibited suitable synergistic effects with intumescent flame retardant on the enhancement of fire resistance of PLA.

**Scheme 1:**
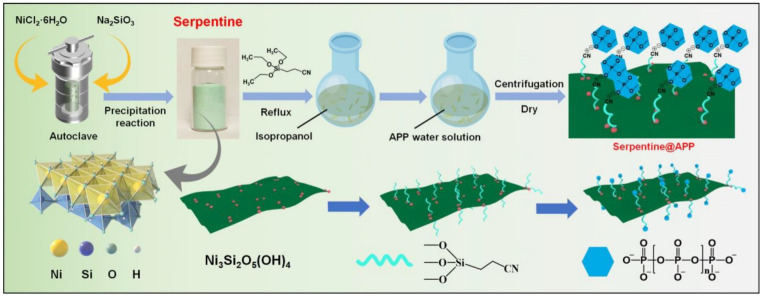
The synthesis route of serpentine@APP.

Serpentine [Ni_3_Si_2_O_5_(OH)_4_] is a type of transition metal silicate hydroxide that exists extensively in the oceanic lithosphere [19]. It is a silicate with unlimited two-dimensional extension made of Si-O tetrahedrons. The oxygen atop the unshared siloxane tetrahedron possesses a residual negative charge (Scheme 1), which can react with Ni^2+^ to produce Ni-O octahedron sheets. Lamellar serpentine nanosheets have been widely utilized for catalysis [20], energy storage [21], and sensors [22]. It is rich in OH groups and has a similar structure to LDH, making it potentially useful in the flame-retardant sector of polymer matrices. However, the application of serpentine as a promising flame retardant in polymer composites is rarely reported. 

In this work, lamellar serpentine nanosheets were synthesized by precipitation reactions under hydrothermal conditions. Then ammonium polyphosphate (APP) was grafted onto the serpentine nanosheets via electrostatic self-assembly by using 2-cyanoethyltriethoxysilane as a chemical bridge. The APP-functionalized serpentine (serpentine@APP) was incorporated into the PLA matrix by melt compounding as a novel type of flame-retardant hybrid filler. The effects of the as-fabricated serpentine@APP on the rheological properties, mechanical properties, and flame-retardant performance of PLA composites were also investigated.

## 2. Experimental

### 2.1. Materials

PLA (Ingeo 4032D) with a density of 1.25 g/cm^3^ was supplied by Natureworks (Plymouth, MN, USA). Nickel chloride hexahydrate and sodium silicate were purchased from Aladdin Bio-Chem Co., Ltd. (Shanghai, China). 2-cyanoethyltriethoxysilane, ammonium polyphosphate (APP, n < 20), and isopropanol were supplied by J&K Co., Ltd. (Beijing, China).

### 2.2. Preparation of Serpentine@APP Hybrid

The synthesis route of ammonium polyphosphate decorated serpentine (serpentine@APP) was shown in Scheme 1. The serpentine Ni_3_Si_2_O_5_(OH)_4_ nanosheets were synthesized by precipitation reactions under hydrothermal conditions [23]. Typically, 1 mmol of Na_2_SiO_3_ and 35 mL of DI water were mixed by vigorous stirring. Then, 1.5 mmol of NiCl_2_ was introduced to the Na_2_SiO_3_ solution to generate light green precipitates. Subsequently, the as-prepared suspension was moved into a 100 mL Teflon-lined stainless steel container and heated in an oven at 180 °C for 12 h. To remove the excess OH^-^, Cl^-^, and Na^+^ ions, the resulting hydrothermal treatment materials were centrifuged and rinsed with DI water and isopropanol, respectively. The washed precipitate was collected after being dried at 120 °C.

1.0 g of as-prepared Ni_3_Si_2_O_5_(OH)_4_ nanosheets were mixed with 200 mL of isopropanol by ultrasonication. Then the solution was treated with 100 μL of 2-cyanoethyltriethoxysilane dropwise for 2 h at 90 °C, followed by centrifugation and rinsing with DI water and isopropanol, respectively. A total of 5.0 g of APP was dissolved in 200 mL of DI water with vigorous stirring to form a clear solution. After the APP was dispersed completely, 1.0 g of silane-modified serpentine was subsequently added with vigorous stirring. Then the mixture was centrifuged at 10,000 rpm for 15 min and dried in a vacuum oven. The obtained APP-functionalized serpentine was denoted as serpentine@APP.

### 2.3. Preparation of PLA Composites

A counter-rotating internal mixer (PLASTIC-ORDER, Brabender, Germany) with a rotation speed of 60 rpm was used to melt compound PLA composites containing 2 wt% different types of fillers for 10 min at 180 °C. Following that, the samples were hot-molded (185 °C, 10 min) into specimens of various sizes. The formulations of PLA composites are shown in Table 1.

### 2.4. Characterization

The morphology of serpentine, serpentine@APP, and PLA composites was observed by scanning electron microscopy (SEM, FEI, Quata 250, Hillsboro, FL, USA). Before the SEM observations, the samples were sputtered with a thin layer of gold. An atomic force microscope (AFM, VEECO Nanoscope, Plainview, NY, USA) was used to measure the thickness and morphology of the synthesized serpentine nanosheets in tapping mode. The Fourier transform infrared (FT-IR) spectra were carried out on a Fourier transform infrared spectrometer (Nicolet, Nexus 670, Ramsey, MN, USA) in the range of 400–4000 cm^−1^. The elemental compositions were analyzed by X-ray photoelectron spectroscopy (XPS, Physical Electronics, PHI 5802 spectrometer, Chanhassen, MN, USA). The thermal stability was investigated by a thermogravimetric analyzer (TGA, Netzsch, TGA-209F3, Selb, Germany) in the temperature range of 30–700 °C under nitrogen flow. The heating rate was fixed at 10 °C/min. The limiting oxygen index (LOI) was measured on an HC-2 oxygen index instrument (Jiangning Analytical Instrument, Jiangning, China). The UL-94 rating of the samples was evaluated by a vertical burning tester (FTT, Derby, UK). The fire characteristics of the PLA composites were measured on a cone calorimeter (FTT, UK) under an external heat flux of 35 kW/m^2^ based on the standard of ISO-5660. The structure of char residues was analyzed by a Raman spectrometer (LabRAM ARAMIS, Villeneuve d’Ascq, Paris, France). The dynamical rheological properties were measured on an Anton-Paar MCR-302 dynamic rheometer (Graz, Austria). A dynamic mechanical analyzer (Netzsch, DMA 242, Weimar, Germany) was utilized to examine the dynamic mechanical properties in tensile mode. The rectangular specimens with a dimension of 30 × 4 × 0.5 mm^3^ were measured in the temperature range of 0–80 °C at a fixed heating rate of 3 °C/min. The tensile properties were evaluated on an electronic testing machine (Type 5566, Instron, Norwood, MA, USA). The dumbbell specimens with 75 × 4 × 1 mm^3^ were tested at a fixed speed of 1 mm/min.

## 3. Results and Discussion

### 3.1. Characterization of Serpentine@APP

**Figure 1:**
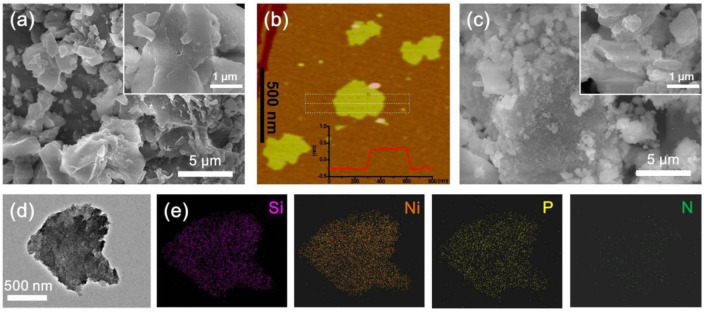
(**a**) SEM images of serpentine, (**b**) AFM image of serpentine (Insert is the height of the selected curve); (**c**) SEM image, (**d**) TEM image, and (**e**) the corresponding EDX elemental mapping of Si, Ni, N, and P elements of serpentine@APP.

The morphology of the prepared serpentine is shown in Figure 1a. The serpentine nanosheets exhibit a layered structure with a smooth surface. The AFM image in Figure 1b confirms the lamellar structure. The height of the region marked by the white line in the AFM image ranges from 0.6 to 0.7 nm, corresponding to the height of monolayer serpentine. As for serpentine@APP, a rougher surface of serpentine@APP is clearly observed in Figure 1c,d, which is due to the charge self-assembling between APP and the silane-modified serpentine. In Figure 1e, the corresponding EDX mapping images confirm the existence of Si, Ni, P, and N elements, which disperses evenly onto the surface of serpentine@APP, indicating APP has been successfully decorated onto the surface of serpentine.

**Figure 2:**
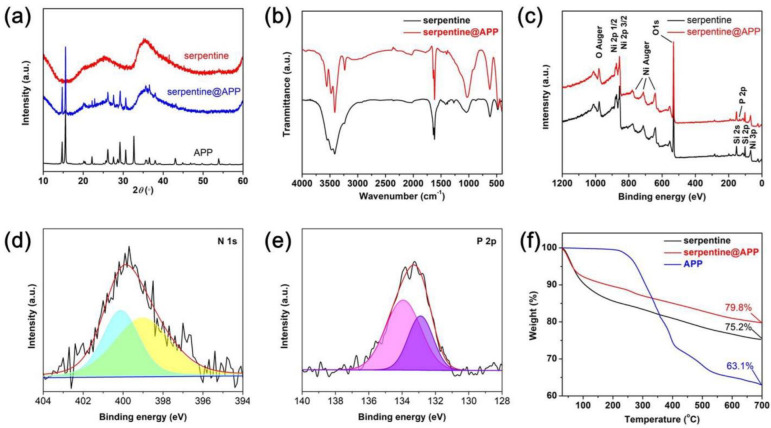
(**a**) XRD patterns (**b**) FT-IR spectra (**c**) XPS full scan spectra of serpentine and serpentine@APP; (**d**) N 1s, (**e**) P 2p high-resolution spectra of serpentine@APP, and (**f**) TGA curves of APP, serpentine, and serpentine@APP.

The XRD patterns of APP, serpentine, and serpentine@APP are depicted in Figure 2a. The four diffraction peaks at 20.4°, 25.3°, 34.1°, and 35.3° are assigned to the (110), (004), (200), and (202) planes of serpentine, respectively, which are consistent with the serpentine card (JCPDS no. 49-1859) [24,25]. Moreover, the diffraction peaks of serpentine are very broad, which is consistent with the nanostructured nature of this material. The XRD patterns indicate that the Ni_3_Si_2_O_5_(OH)_4_ nanosheets were synthesized successfully. There are several small diffraction peaks between 20° and 31° for serpentine@APP, which are attributed to the grafted APP. In addition, the diffraction peak at 32.7° in APP disappears in the pattern of serpentine@APP, suggesting there is a chemical interaction between APP and serpentine. The FT-IR spectra of serpentine and serpentine@APP are shown in Figure 2b. The sharp peaks at 3413 cm^−1^ and 1618 cm^−1^ are generated by the -OH vibration in Ni_3_Si_2_O_5_(OH)_4_. The characteristic peak at 1042 cm^−1^ is owing to the Ni–O–Si chemical bond [26]. The band at 619 cm^−1^ corresponds to the Ni–O bond. In addition, the peak at 484 cm^−1^ is attributed to the symmetric stretching vibration of the Si–O bond [27]. These characteristic peaks further prove the formation of nickel silicate in the hybrid. The chemical compositions of serpentine and serpentine@APP were evaluated by XPS, and the corresponding data are shown in Figure 2c–e. In Figure 2c, the presence of Ni, Si, C, and O is clearly observed in the full scan of the serpentine. The appearance of C may be attributed to pollution from the environment. The high-resolution spectra of N 1s and P 2p of serpentine@APP in Figure 2d,e confirm that the APP was attached to the serpentine successfully. To measure the thermal stability of APP, serpentine, and serpentine@APP under a nitrogen atmosphere, the TGA curves are shown in Figure 2f, and the corresponding TGA data are shown in Table S1. The pure APP begins to degrade (*T*_10_, the temperature that corresponds to 10% weight loss) at 309.4 °C and to decompose most rapidly (*T*_max_, the temperature that corresponds to the maximum weight loss rate) at 357.1 °C. As for serpentine and serpentine@APP, both of them exhibit a quick decomposition stage below 100 °C, which is attributed to the loss of absorbed water. With the decoration of APP, the *T*_10_ for serpentine@APP increases from 106.5 to 182.1 °C as compared with pure serpentine. It is because the decorated APP may serve as a protective coating, delaying the decomposition of the serpentine [28]. These above results indicated that serpentine@APP had better thermal stability than pure serpentine.

### 3.2. Fractured Surface Analysis of PLA Composites

**Figure 3:**
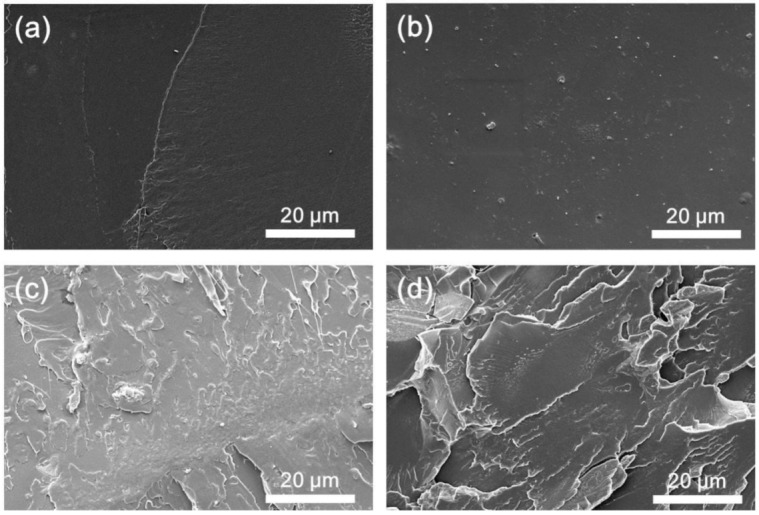
SEM images of the cryo-fracture surfaces of (**a**) pure PLA, (**b**) PLA/APP, (**c**) PLA/serpentine, and (**d**) PLA/serpentine@APP composites.

The morphology of the cryo-fractured surfaces of the pure PLA and its composites is shown in Figure 3. It is evident that pure PLA has a smooth fractured surface (Figure 3a) due to its brittle fracture at low temperatures. In Figure 3b, some APP particles are exposed on the smooth surface of the PLA/APP composite. With the incorporation of serpentine, the PLA/serpentine composite (Figure 3c) exhibits a much rougher surface than that of pure PLA. That is because the presence of rigid serpentine fillers can absorb more energy and inhibit crack propagation during fracture. As for the PLA/serpentine@APP composite (Figure 3d), the introduction of serpentine@APP also increases the cracks in the surface. In addition, there are no cavities on the surfaces of PLA/serpentine and PLA/serpentine@APP composites, indicating that both serpentine and serpentine@APP have suitable interfacial interaction with the PLA matrix.

### 3.3. Thermal Stability

**Figure 4:**
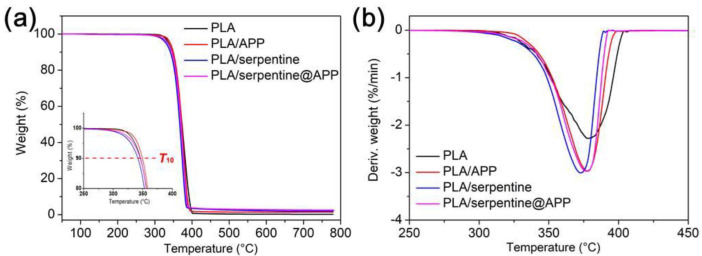
Thermal stability of pure PLA and its composites, (**a**) TGA, (**b**) DTG.

The thermal stability of PLA composites was evaluated by TGA, as shown in Figure 4. The temperatures at 10% weight loss (*T*_10_), the temperatures at the maximum weight loss rate (*T*_max_), and the char residues at 800 °C are listed in Table 2. All of the samples in Figure 4a show a single primary decomposition stage between 300 and 400 °C, which corresponds to random chain scission and particular chain-end scission. [29]. With the incorporation of APP, the *T*_10_ of the PLA/APP composite increases from 346.2 to 349.7 °C as compared with pure PLA. However, the incorporation of serpentine results in a slight decrease in *T*_10_. It is speculated that serpentine containing a large number of hydrogen groups has lower thermal stability, as shown in Figure 2f. After decoration with APP, the *T*_10_ of the PLA/serpentine@APP shifts to a higher value (346.0 °C). The *T*_max_ for pure PLA, PLA/APP, PLA/serpentine, and PLA/serpentine@APP in Figure 4b is 379.0, 377.1, 373.5, and 376.7 °C, respectively. As shown in Table 2, almost no char residue is observed for the pure PLA at 800 °C. Meanwhile, the presence of APP and serpentine can increase the char residue of PLA composites. The increased char residue weight can contribute to the enhancement of fire safety for PLA composites.

### 3.4. Flame-Retardant Performance

The limited oxygen index (LOI) and UL-94 test results are summarized in Table 2. It is clear that pure PLA has an LOI value of 19 and no rating in the UL-94 test, indicating it is a flammable material. With the addition of serpentine@APP, the LOI value of PLA composite increases from 19 to 25.5 but can only achieve a UL-94 V-1 rating. However, all of the samples have dripping phenomena due to their low melting viscosity.

**Figure 5:**
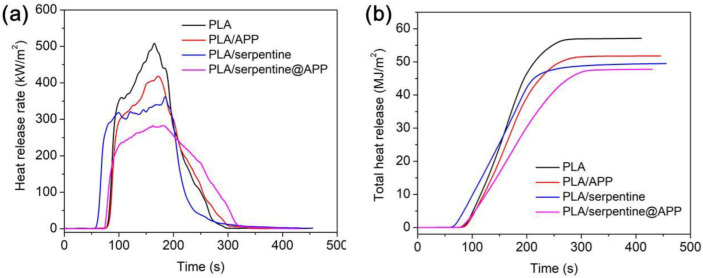
Cone calorimeter tests of pure PLA and its composites. (**a**) HRR, (**b**) THR.

A cone calorimeter is a useful tool to evaluate the fire safety of polymers in real fire accidents [30]. The heat release rate (HRR), total heat release (THR), and char residues of PLA composites as a function of time are presented in Figure 5, and more detailed parameters, including time to ignition (TTI), peak heat release rate (pHRR) are summarized in Table 3. In Figure 5a, pure PLA exhibits a pHRR value of 507.8 kW/m^2^ and a THR value of 57.1 MJ/m^2^. With the addition of APP, the pHRR of PLA/APP decreases to 419.7 kW/m^2^, which is decreased by 17.3% as compared to pure PLA. This is because the thermal decomposition of APP can release free radical scavengers (·P and ·PO), which may trap flammable radicals (·O, ·H, and ·OH), inhibit the burning reaction chain, and generate more solid products [31]. As for PLA/serpentine, the presence of serpentine can serve as a thermal barrier to prolong the escape paths for the flammable gases. In addition, the nickel element in serpentine has a suitable catalytic carbonization effect that promotes the formation of the char layer [32]. The PLA/serpentine@APP exhibits a pHRR value of 284.7 kW/m^2^ and a THR value of 47.8 MJ/m^2^, which are reduced by 43.9% and 16.3%, respectively, as compared to those of pure PLA. In Figure 5b, it is clear that the slope of PLA/serpentine@APP is lower than those of other curves, indicating that the combination of APP and serpentine has suitable synergistic flame-retardant effects that suppress heat release during combustion.

### 3.5. Char Residues Analysis

**Figure 6:**
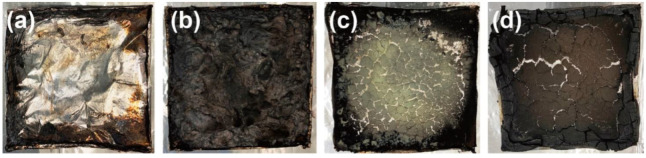
Digital images of char residues of the PLA samples after cone tests. (**a**) pure PLA, (**b**) PLA/APP, (**c**) PLA/serpentine, and (**d**) PLA/serpentine@APP.

**Figure 7:**
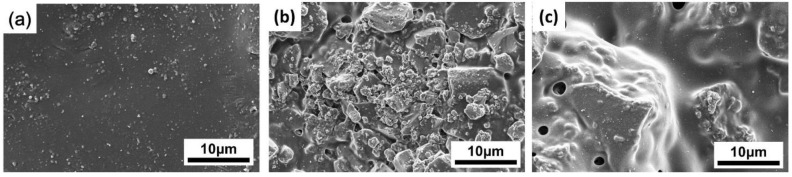
SEM images of the char residues of (**a**) PLA/APP, (**b**) PLA/serpentine, and (**c**) PLA/serpentine@APP.

Figure 6 shows the photographs of char residues of PLA composites after the cone calorimeter test. It is noted that pure PLA has been completely burned, and very little char residue can be found in the aluminum foil. Meanwhile, the char residue of PLA/serpentine shows a fluffy and discontinuous state. With the addition of APP, an extremely thin and dense carbon layer is formed on the sample of PLA/APP after burning and adhering to the tin foil at the bottom. Moreover, the char residue of PLA/serpentine@APP is relatively continuous, and a large carbon block is formed on the surface. To investigate the microstructure of char residues, the SEM images of the char residues are shown in Figure 7. PLA/APP has a compact and continuous char layer in Figure 7a due to the presence of phosphorus derivatives in APP. It is observed that the lamellarly structured serpentine of PLA/serpentine is loosely distributed after combustion, and the area of the continuous carbon layer is small (Figure 7b). While the serpentine in the char residue of PLA/serpentine@APP is bonded together to form an effective carbon layer, which acts as a barrier to the diffusion of O_2_ and heat, preventing further combustion of the substrate. A small amount of pores appears in the carbon layer of PLA/serpentine@APP (Figure 7c), which may be caused by the release of incombustible gases (CO, CO_2_, and NH_3_) into the gas phase by APP before the decomposition of the PLA matrix during the combustion process [33]. The presence of serpentine@APP contributes to the formation of more dense and complete carbon layers, which can more effectively prevent the exchange of heat and pyrolysis gas products with the external area [34], thereby delaying and restricting the development of combustion to achieve the flame-retardant effect.

**Figure 8:**
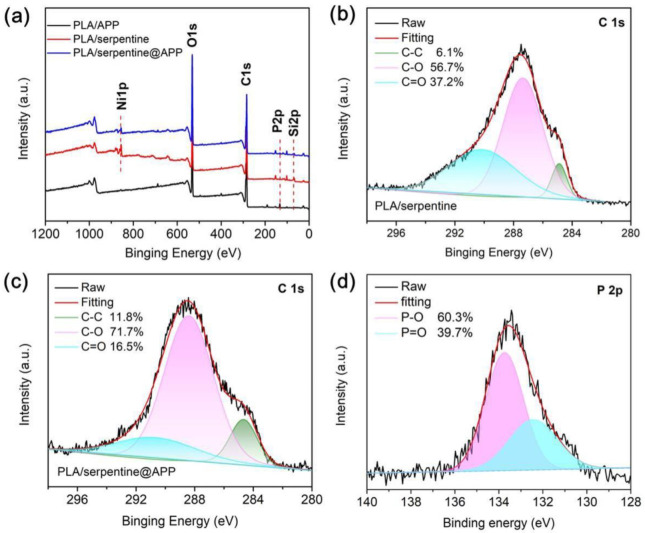
XPS spectra of the char residues after cone calorimeter test. (**a**) Full scanning spectra, (**b**) C 1s spectrum of PLA/serpentine; (**c**) C 1s, and (**d**) P 2p spectra of PLA/serpentine@APP.

The chemical composition of the char residues after the cone calorimeter test was analyzed by XPS, as shown in Figure 8. The XPS full scan spectra in Figure 8a confirm the existence of C, O, Ni, P, and Si elements in the char residues. In Figure 8b,c, the high resolution of the C 1s XPS spectra reveals that the peaks of the C=O (290.7 eV), C–O (288.3 eV), and C–C (284.8 eV) bonds can be fitted separately. Furthermore, the content of C=O and C–O bonds in the carbon residue of the PLA/serpentine sample is lower than that of the PLA/serpentine@APP sample. The molten flow of the PLA matrix during combustion breaks up the carbon layer formation, leading to more contact with air and promoting a more complete combustion of PLA/serpentine. However, it is difficult for the APP to flow with the PLA matrix melt in the PLA/serpentine@APP sample. Meanwhile, it will accelerate the dehydration of the matrix, which is conducive to the rapid formation of a preliminary complete carbon layer after combustion, thereby reducing the contact with O_2_, promoting the incomplete combustion of the matrix, and increasing the content of C=O and C–O [35]. The high resolution of the P 2p spectrum in Figure 8d further confirms the presence of phosphorus groups in PLA/serpentine@APP. The peaks of the P–O (133.7 eV) and P=O bond (132.4 eV) can be fitted separately [36]. Previous studies have demonstrated that the accumulation of phosphorus-containing compounds on the surface of the carbon layer will reduce the permeability of the carbon layer, which is more conducive to improving the barrier properties of the carbon layer [37].

**Figure 9:**
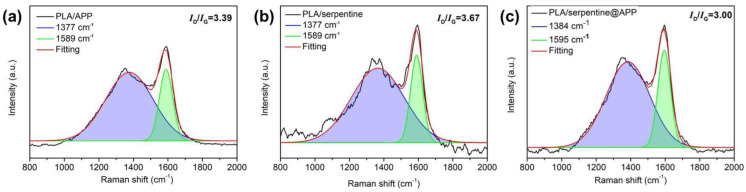
Raman spectra of the char residues of (**a**) PLA/APP, (**b**) PLA/serpentine, and (**c**) PLA/serpentine@APP.

The graphitization degree of the char residues was analyzed by Raman spectra [38]. The two broad bands around 1380 and 1590 cm^−1^ in Figure 9 correspond to the disordered carbon structure of the D band and the graphitized structure of the G band, respectively [39,40]. The integral strength ratio (*I*_D_/*I*_G_) of the D band and the G band can measure the degree of graphitization. The lower the ratio, the higher the degree of graphitization of the carbon layer [41,42]. It is noted that the *I*_D_/*I*_G_ of char residues of PLA/serpentine@APP is 3.00 (Figure 9a), which is lower than those of PLA/serpentine (*I*_D_/*I*_G_ = 3.67, Figure 9b) and PLA/APP (*I*_D_/*I*_G_ = 3.39, Figure 9c). The results show that the APP-modified serpentine can obtain a carbon layer with a higher degree of graphitization than the serpentine alone. APP will form pyrophosphoric acid or polyphosphoric acid in the process of thermal decomposition [43], which will destroy the PLA bone chain and the hydroxyl group in the matrix to form a higher-quality carbon layer. The highly graphitized carbon structure layer can act as a flame-retardant barrier that keeps the fire from spreading during the combustion process of the substrate.

### 3.6. Rheological Behavior of PLA Composites

**Figure 10:**
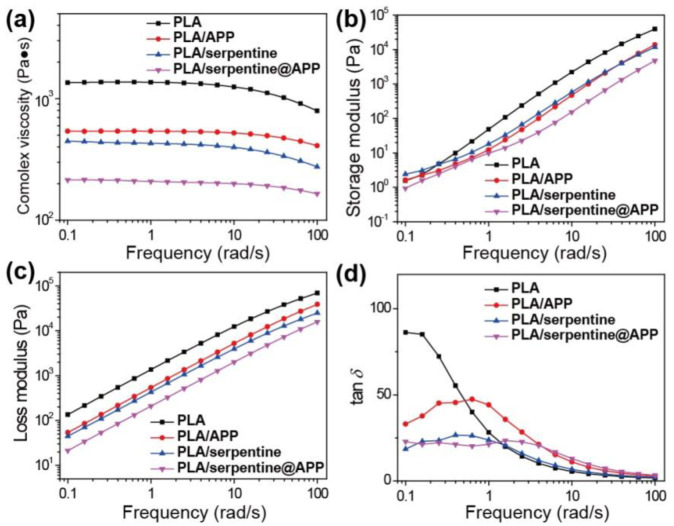
Dynamical frequency sweep of PLA composites. (**a**) complex viscosity, (**b**) storage modulus, (**c**) loss modulus, and (**d**) loss factor.

The linear viscoelastic region of pure PLA and its composites was determined by the dynamical strain sweep tests [44,45], as depicted in Figure S1. All of the materials show a linear viscoelastic domain up to 30% strain. The apparent shear-thinning phenomena occur when the shear amplitude is above 30% strain. Therefore, the dynamical frequency sweep experiment was carried out using a strain amplitude of 1% to avoid non-linear behavior at low frequencies [46]. The complex viscosity (η*), storage modulus (*G*’), loss modulus (*G*’’), and damping factor (tan*δ*) as a function of frequency are shown in Figure 10. It is noted that a Newtonian plateau in the low-frequency region is observed for all of the samples in Figure 10a, followed by a shear-thinning tendency at the high-frequency region. In addition, the shear-thinning phenomenon of pure PLA is more profound than that of PLA/sepentine@APP, indicating that the presence of sepentine@APP contributes to the disentanglement of PLA chains. Interestingly, it is noted that the addition of serpentine will result in a decrease in the η* of PLA composites. It can be attributed to the plasticizing effect of the 2D serpentine, which can decrease the rectangle of the polymer chains. In Figure 10b,c, the *G*’ and *G*’’ show a similar decrease tendency. However, the PLA composite containing serpentine has a greater storage modulus at low frequencies and a more pronounced decline at high frequencies. This phenomenon may be attributed to the 2D serpentine flake being oriented at a high rotation speed, which is consistent with other 2D materials. Interestingly, the PLA/sepentine@APP composite has a much lower storage modulus than that of APP or serpentine applied solely in the high-frequency region. It is speculated that the low molecular weight APP has a suitable synergistic plasticizing effect with serpentine. The value of tan*δ* represents the ratio between *G*’ and *G*’’, which indicates the dissipation of heat energy. In Figure 10d, the values of tan*δ* are decreased obviously with the addition of serpentine and sepentine@APP at the low-frequency region. This is because the presence of serpentine or sepentine@APP contributes to the elasticity improvement of PLA.

### 3.7. Mechanical Properties of PLA Composites

**Figure 11:**
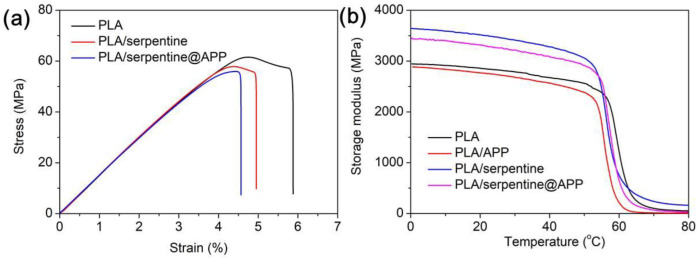
(**a**) Tensile stress versus strain of tensile test, (**b**) storage modulus of dynamical mechanical analysis of pure PLA and its composites.

Figure 11a displays the tensile stress versus strain curves of PLA composites, and the relevant data are provided in Table 4. In Figure 11a, all of the samples display the typical brittle behavior that breaks at certain places quickly after the yielding point. The pure PLA has a maximum tensile stress of 59.4 MPa, and an elongation at break of 6.05%. The stiffness of serpentine is considered to be the reason why the Young’s modulus of PLA/serpentine jumps from 1476.6 to 1553.3 MPa when compared to pure PLA. However, the mechanical properties of PLA/serpentine@APP somewhat deteriorate when compared to PLA/serpentine. That is because the APP has a small molecular weight and is grafted onto the surface of the serpentine by electrostatic interaction, leading to reduced tensile strength and elongation at break. As shown in Figure 11b, the storage modulus of PLA/APP is lower than that of pure PLA in the whole temperature range. Accordingly, it can be explained by the fact that the addition of APP has a plasticizing effect on PLA.

## 4. Conclusions

In this study, serpentine Ni_3_Si_2_O_5_(OH)_4_ was first synthesized by precipitation reactions under hydrothermal conditions. Then APP was electrostatically grafted onto the surface of serpentine to create a novel type of hybrid flame-retardant additive (serpentine@APP), followed by mixing with PLA by melt compounding. The PLA composite containing 2 wt% serpentine@APP exhibited suitable fire safety enhancement with a 43.9% reduction in pHRR and a 16.3% reduction in THR as compared to those of pure PLA. This is because the decomposition of APP will release free radical scavengers, the thermal barrier effect of serpentine, and the catalytic carbonization effect of the nickel element in serpentine. The presence of serpentine@APP could reduce the complex viscosity of PLA, which improved its processability. In addition, the tensile stress and elongation at the break of PLA/serpentine@APP showed a small decrease as compared to pure PLA. This research offers a feasible strategy to develop a flame-retardant hybrid filler to increase the fire safety of PLA.

## Figures and Tables

**Table 1:** Formulations of pure PLA and its composites.

Sample	PLA (wt%)	APP (wt%)	Serpentine (wt%)	Serpentine@APP (wt%)
PLA	100	--	--	--
PLA/APP	98	2	--	--
PLA/serpentine	98	--	2	--
PLA/serpentine@APP	98	--	--	2

**Table 2:** TGA data for pure PLA and its composites.

Samples	*T*_10_ (°C)	*T*_max_ (°C)	Residues at 800 °C (wt%)
PLA	346.2	379.0	0.21
PLA/APP	349.7	377.1	1.39
PLA/serpentine	341.5	373.5	1.72
PLA/serpentine@APP	346.0	376.7	1.95

**Table 3:** LOI, UL-94 test, and cone calorimeter data for pure PLA and its composites.

Samples	LOI (%)	UL-94 Rating	Dripping	TTI (s)	PHRR (kW/m^2^)	THR (MJ/m^2^)
PLA	19	NR	Yes	75	507.8	57.1
PLA/APP	22	V-2	Yes	77	419.7	51.8
PLA/serpentine	23.5	V-2	Yes	68	362.4	49.5
PLA/serpentine@APP	25.5	V-1	Yes	72	284.7	47.8

**Table 4:** Tensile properties for pure PLA and its composites.

Samples	Tensile Stress (MPa)	Young’s Modulus (MPa)	Elongation at Break (%)
PLA	59.4 ± 2.3	1476.6 ± 74.1	6.05 ± 0.49
PLA/serpentine	57.9 ± 2.8	1553.3 ± 73.9	4.97 ± 0.56
PLA/serpentine@APP	56.6 ± 1.5	1493.2 ± 44.3	4.83 ± 0.21

## Data Availability

The raw/processed data generated in this work are available upon request from the corresponding author.

## Supplementary Materials

The following supporting information can be downloaded at https://www.mdpi.com/article/10.3390/polym14235255/s1, Figure S1: Strain sweep of pure PLA and its composites; Table S1: TGA data of APP, serpentine and serpentine@APP.
