# Supplementary material for: Self-Assembled Serpentine Ni3Si2O5(OH)4 Hybrid Sheets with Ammonium Polyphosphate for Fire Safety Enhancement of Polylactide Composites
Source: Polymers (Basel). 2022 Dec 1;14(23):5255. doi: 10.3390/polym14235255 (PMC9741248; doi:10.3390/polym14235255)
Supplement: Supplementary file 1 [file polymers-14-05255-s001.zip › polymers-2054984-supplementary.pdf]

Supporting Information

Self-assembled serpentine $Ni_3Si_2O_5(OH)_4$ hybrid sheets with ammonium polyphosphate for fire safety enhancement of polylactide composites

Xiaohong Yi [a], Jingshu Huang [b], Yizhang Tong [b], Hui Zhao [c,*], Xianwu Cao [b], Wei Wu [a,b,*]

[a] Jihua Laboratory, Foshan 528200, China.

[b] Key Laboratory of Polymer Processing Engineering of Ministry of Education, South China University of Technology, Guangzhou 510640, China.

[c] Guangxi Key Laboratory of Calcium Carbonate Resources Comprehensive Utilization, College of Materials and Chemical Engineering, Hezhou University, Hezhou City 542899, China.

* Correspondence: wuwei@jihualab.ac.cn (W. Wu), zhh@gxu.edu.cn (H. Zhao)

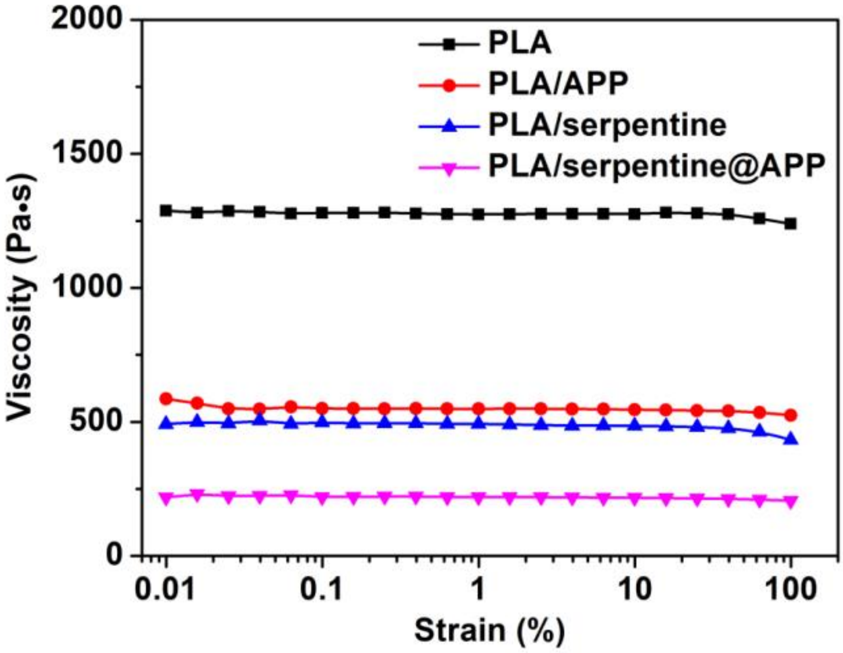

Figure S1. Strain sweep of pure PLA and its composites.

Table S1. TGA data of APP, serpentine and serpentine@APP.

Samples	T_{10} (°C)	T_{max} (°C)	Residue at 700 °C (wt%)
APP	309.4	357.1	63.1
serpentine	106.6	56.8	75.3
serpentine@APP	182.1	50.9	79.8
